# What is the efficacy and effectiveness of telemedicine intervention for deaf signing populations in comparison to face-to-face interventions? A systematic review

**DOI:** 10.1186/s12913-023-09509-1

**Published:** 2023-06-22

**Authors:** Katherine Rogers, Karina Lovell, Alys Young

**Affiliations:** The University of Manchester, Manchester, England

**Keywords:** Sign language, Telemedicine, Face-to-face, Systematic review

## Abstract

**Background:**

Deaf signing populations face inequality in both access to health services and health outcomes. Telemedicine intervention might offer a potential solution to address these inequalities in mental health and health related services, therefore a systematic review was carried out. The review question was: “What is the efficacy and effectiveness of telemedicine intervention for Deaf signing populations in comparison to face-to-face interventions?”.

**Methods:**

The PICO framework was applied to identify the components of the review question for this study. The inclusion criteria were: Deaf signing populations; any intervention that includes the delivery of telemedicine therapy and/or the delivery of assessment (e.g. psychological assessments) using telemedicine; and any evidence for the benefits, efficacy and effectiveness of telemedicine intervention with Deaf people whether in health and/or mental health services. The databases PsycINFO, PubMed, Web of Science, CINAHL, and Medline were searched up to August 2021.

**Results:**

Following the search strategy, and after the duplicates were removed, 247 records were identified. Following screening, 232 were removed as they did not meet the inclusion criteria. The remaining 15 full-text articles were assessed for eligibility. Only two met the criteria to be included in the review (both concerned telemedicine and mental health interventions). However, they did not fully answer the review’s research question. Therefore, the evidence gap remains regarding the effectiveness of telemedicine intervention for Deaf people.

**Conclusions:**

The review has identified a gap in the knowledge on the efficacy and effectiveness of telemedicine intervention for Deaf people when compared with face-to-face interventions.

## Background

In comparison with the hearing population, Deaf people who use sign language experience poorer mental health [1], poorer health-related outcomes (e.g. obesity; cardiovascular disease; and diabetes mellitus) [2], as well as experiencing inequality in accessing services [3]. For example, Kvam et al. [1] found that the prevalence of anxiety and depression amongst Deaf adults was 33.8%, compared to 15% for hearing populations [4]. The impact of this can also be seen in a wider social context, for example, in the cost to the economy of inappropriate support for Deaf people who are experiencing mental health difficulties [5]. Primary mental health care poses a particular challenge. In the United Kingdom (UK), the number of available psychological therapists trained to work both within and outside of the NHS (National Health Service) talking therapies (Improving Access to Psychological Therapies (IAPT) programme), and are able to meet the cultural and linguistic needs of Deaf people, are few, and Deaf-specialist primary mental health services are not available nationally in the UK [6]. However, in March 2022 the NHS England awarded SignHealth, a specialist service for Deaf people, a contract to deliver IAPT service in British Sign Language (BSL) to Deaf people throughout England [7]. In response to the Covid-19 pandemic, services were rapidly forced to accept this medium of telemedicine to deliver mainstream services, although not without some criticism.

Telemedicine, in this case the use of videoconferencing for Deaf people, potentially offers creative and accessible solutions for addressing a range of health-related matters amongst communities who use a signed language because of its visual modality. Like any other sign languages (e.g. American Sign Language (ASL)), in the UK, BSL is the language of the Deaf community and BSL is a complete visual language in its own right that uses space, location, handshape and movement to convey meaning [8]; consequently there is no written form of the language. Additionally, members of these signing communities are usually geographically dispersed. For many Deaf people all over the world, being Deaf is not about being unable to hear but is about a sense of culturo-linguistic identity as they see themselves primarily as a language minority rather than as a disabled group [9]. When one thinks of a community, it is easily assumed that they live in the same place, however this is not the case with signing communities, as there are no places where the majority of inhabitants are Deaf [10]. There are, however, some places in which there are more Deaf inhabitants per capita such as Derby in the UK [11], and Rochester, New York for the USA [12].

Videoconferencing in many aspects of health (diagnosis, assessment, treatment, recovery) in the general populations has become more widespread in the 21st century as online digital technologies and capabilities have improved [13]. In the Deaf community, videoconferencing has been used as a medium for communication to support the prevention and treatment of medical conditions such as substance misuse [14], and more generally for accessing health services either directly in a signed language or through a sign language interpreter. For example, BSL Health Access, was launched in the UK in 2020 although closed in 2021 due to insufficient funding (https://bslhealthaccess.co.uk/). Psychological assessments have been carried out with Deaf people online via video format (e.g. Belk et al. [15] for the asynchronous psychological assessment). Although not the focus of this review, Video Remote Interpreting (VRI) is becoming more common in health services especially in the US and Canada despite concerns that the use of VRI has decreased patient/provider engagement [16] and that clinical encounters are hampered by poor connectivity [17]. However, telemedicine is an under-developed area of study as the efficacy of the delivery medium and its effectiveness regarding health improvements in general, and psychological interventions in particular, have not yet been tested with Deaf sign language users.

In the context of mental health in relation to hearing populations, it has been found that telemedicine is as effective as face-to-face therapy [18]. A Cochrane review undertaken by Flodgren et al. [19], reported no difference in the clinical outcomes between videoconferencing and face-to-face therapy. Deaf people were not included in these studies. A systematic review with specific reference to the context of Deaf populations, in relation to health and mental health telemedicine interventions, is needed in order to assess and synthesise the current knowledge. This will establish a better understanding of the current knowledge with respect to telemedicine interventions for Deaf populations, which would inform healthcare professionals and commissioners, which would in turn assist them in tackling the inequalities and issues faced by Deaf people. Searches from PROSPERO and the Cochrane library have identified that there are no existing, nor current, systematic reviews on the topic of telemedicine intervention for Deaf populations. Scoping searches for published articles (non-systematic reviews), on the topic of telemedicine interventions for Deaf populations, identified existing published articles in the area of telemedicine and Deaf signing populations (e.g. Gournaris [20]; and Austen & McGrath [21]). This systematic review focused on the efficacy and effectiveness of telemedicine intervention in the field of health and mental health, including remotely delivered psychological assessments and therapeutic interventions, for Deaf signing populations on an international basis in comparison to face-to-face intervention.

## Method

### Review question

What is the efficacy and effectiveness of telemedicine intervention for Deaf signing populations in comparison to face-to-face interventions?

### Inclusion and exclusion criteria

The PICO (Population, Intervention, Comparison, and Outcome) framework [22] was applied to identify the components of the review question for this study. Details of the criteria for inclusion and exclusion for the review are as follows:

#### Population / participants

Given that Deaf people across countries face similar inequality issues (e.g. mental health and health-related outcomes and barriers to accessing mental health services), any studies that involved Deaf signing populations, whether British Sign Language (BSL); American Sign Language (ASL), German Sign Language (Deutsche Gebärdensprache DGS), etc., were included in this review. Any deaf populations who do not use sign language, have been excluded from this review. Any publications that were published in languages other than English and a Signed Language (e.g. published in Deaf Studies Digital Journal) have been excluded from this review.

#### Intervention

Any intervention with Deaf signing populations that included the delivery of telemedicine therapy (whether for health or mental health) and/or the delivery of an assessment (e.g. of recovery or of psychological assessment) using telemedicine in sign language have been included in this review. Examples of this would be if the intervention used a video call to deliver therapy to Deaf people, the use of an asynchronous video health assessment online would be another example. Studies of interventions that used a remote interpreting service (e.g. with the client and health professionals present in the same room but communicating through a remote interpreter) have been excluded from this review.

#### Comparison

Ideally, the usual standard of care/treatment via face-to-face/in person without the use of telemedicine would be the comparator. However, because of the limited research available on the topic of telemedicine involving Deaf signing populations, a comparator approach with studies which involved having a face-to-face/in person as a control group has not been used.

#### Outcome(s)

Any evidence of benefit (including acceptability), efficacy and effectiveness have been included in this review. Examples of benefit, efficacy and effectiveness include: the satisfaction with the use of telemedicine from a Deaf person’s perspective; measures of mental health outcomes (e.g. the Patient Health Questionnaire 9-item scale (PHQ-9)) score) and the demand for a telemedicine service for Deaf people.

### Search strategy

PsycINFO, PubMed, Web of Science, CINAHL, and Medline, and the grey literature sources (e.g. conference presentations, and reports), the relevant investigators working in the field of Deaf populations were also contacted and were searched for in this review study up to August 2021. Forward citation sources, from the relevant reference lists, were also searched for completeness (the method is described in the review protocol for a separate study by the same first two authors [23]. Table 1 shows a list of search terms for this review. The results from the fifth search (S5) were then moved to EndNote, to begin the screening stage.

**Table 1:** Search terms used

#	
S1	(deaf* OR “hearing impair*” OR “hearing loss”) AND (“sign* language” OR signing)
S2	(telemedicine OR telemental health OR telehealth OR telepsyc* OR telepractice OR telecomm* OR teleinterv*) OR (video*)
S3	(assessment OR instrument* OR measure* OR questionnaire OR survey)
S4	(efficacy OR effective*) OR (reliab* OR valid*) OR (satisf* OR accept*)
S5	S1 AND S2 AND S3 AND S4

#### Screening and selection

**Fig. 1:**
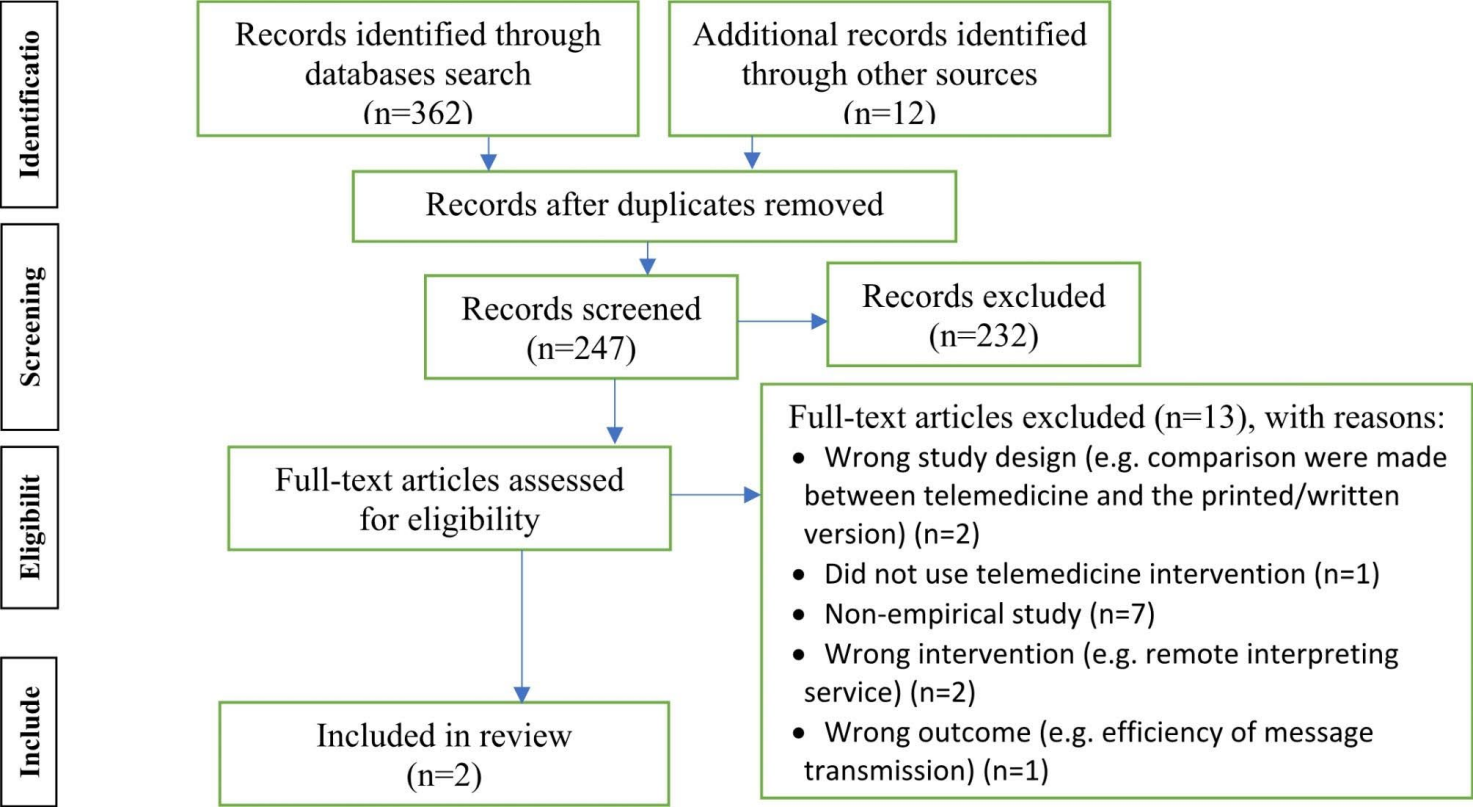
PRISMA flow diagram: the findings from the searches

The selection of the studies to be included in the review involved two stages: (i) screening of the title and abstract; and (ii) full text review (see Fig. 1 for PRSIMA flow diagram).

### Quality assessment

Quality assessment followed the framework tool developed by Critical Appraisal Skills Programme [24] (CASP checklists). The CASP checklists were considered suitable as they included both quantitative and qualitative studies. The type of CASP checklist used for a study depends on the type of study (https://casp-uk.net/casp-tools-checklists/). Examples of questions used to assess the quality of a study included; study validity, what the results were, and whether the findings were useful. To reduce the risk of bias in the review of assessing the quality of each study, two reviewers (AUTHOR ONE AND TWO) undertook the full text review stage. A summary of the quality assessment findings for each study is presented in Table 2.

### Data extraction

The relevant data from each study were extracted from the full-text articles, and recorded in a Microsoft Excel document. The descriptive data (e.g. methods; participant characteristics; setting; and interventions) and analytical data (i.e. outcome data) to be extracted were included, for example: author(s); year of publication; publication type; study ID; country where the study was carried out; study design; number of participants; age; gender; inclusion/exclusion criteria; intervention; and comparisons (if using a control group); outcome data/results (statistical data or results from qualitative data).

## Results

As a result of the databases searched 362 records were identified, which were combined with additional records identified through other sources (n = 12). Duplicate records were identified (N = 127) resulting in 247 records. During the title/abstract screening stage 232 records were excluded which left 15 full-text articles assessed as eligible. The review of full text articles (n = 15) resulted in 13 articles being excluded, leaving only two to be included in the review (see Fig. 1 for PRISMA (Preferred Reporting Items for Systematic Reviews and Meta-Analyses) flow diagram). The two articles included for the data extraction process of the systematic review both related to telemedicine mental health interventions.

### Description of the studies included

The two included articles were Crowe et al. [25] and Pertz et al. [26]. The study by Crowe et al. [25], which was published in a peer-review journal, was carried out in Maryland, USA where the two community health clinics that took part in this study are based. The study used a pre- and post-test design and a group comparison design. As such, the participants were grouped into either: (a) control group (face-to-face psychotherapy); and (b) experimental group (telepsychiatry). The inclusion and exclusion criteria were not stated but all participants were recruited from either of the two specialist mental health services for Deaf people in Maryland. The intervention is telepsychiatry using videoconferencing equipment with a therapist delivered in American Sign Language (ASL), although it is not known whether the ASL therapist was Deaf or not. The comparator is traditional face-to-face psychotherapy, with a therapist who is fluent in ASL, although it is not clear how participants were allocated to the face-to-face group (N = 11, 45.8%) or the telepsychiatry group (N = 13, 54.2%). In total, 24 Deaf or hard of hearing participants took part in this study, of which 13 were women (54.2%) and 11 men (45.8%), and the age range of participants was 23–83 years old with the mean age being 46.38 years old (SD = 14.50). The majority of the participants (91.7%) reported that they preferred to use ASL as their primary language. The outcome findings from Crowe et al. highlighted that there were no significant differences in coping abilities and psychiatric symptoms (as measured by the Maryland Outcomes Measurement System Adult Questionnaire (OMS)) between those who used face-to-face psychotherapy and those receiving telepsychiatry. It is not known whether the measurements such as OMS were available in ASL nor that it has been validated to use with the Deaf signing population. Both groups reported a high level of satisfaction with regards to service provision, as measured using Maryland’s Consumer Perception of Care Survey.

The study by Pertz et al. [26], published in a peer-review journal highlighted that its aim was to pilot an accessible, integrated mental health programme for Deaf signers in Michigan, USA including assessing the feasibility of telemental health services. The second focus was on the level of patient acceptance of a telemedicine form of delivery for Deaf patients. The inclusion and exclusion criteria were not stated, but all patients were Deaf ASL users (aged 18 or above) who used family medicine and clinical social work services. The interventions were tailored to each individual patient. Telemental health services were provided by the Clinical Social Worker who was hearing and fluent in ASL, typical in-person visits continued to be available to all patients at their request. The decision to use telemental health or in-person visits was left to the patient to decide, however it was not clear what the comparators were. In total, 50 Deaf patients took part in the integrated program, of whom 40 saw both a family physician (Deaf ASL fluent) and the social worker (hearing ASL fluent), the rest of the participants (n = 10) saw either the family physician or the social worker. There were 24 male (48%) and 26 female (52%) participants, with a range of ages from 18 to 61 plus years old (mean age = 46.4%, SD = 14.2). While the PHQ-9 and GAD-7 (Generalized Anxiety Disorder 7-Item Scale) were used to assess outcomes, there was no comparison between those who used in-person vs. telemental health visits. With regards to the feedback on the use of telemental health services, the option of having an opportunity to receive telemental health care remotely was praised. Two participants who had lower satisfaction scores struggled with video quality issues.

### Study appraisal

Table 2 provides a list of CASP questions that were used in the appraisal of the two studies included in the review.

**Table 2:** CASP appraisal for the studies included in the review

		Crowe et al. (2016)	Pertz et al. (2018)
CASP Q1.	Did the study address a clearly focused research question?	Y	Y
CASP Q2.	Was the assignment of participants to interventions randomised?	CT	N
CASP Q3.	Were all participants who entered the study accounted for at its conclusion?	N	CT
CASP Q4a.	Were the participants ‘blind’ to intervention they were given?	N	N
CASP Q4b.	Were the investigators ‘blind’ to the intervention they were giving to participants?	N	N
CASP Q4c.	Were the people assessing/analysing outcome/s ‘blinded’?	CT	CT
CASP Q5.	Were the study groups similar at the start of the randomised controlled trial?	N	CT
CASP Q6.	Apart from the experimental intervention, did each study group receive the same level of care (that is, were they treated equally)?	CT	CT
CASP Q7.	Were the effects of intervention reported comprehensively?	CT	N
CASP Q8.	Was the precision of the estimate of the intervention or treatment effect reported?	N	N
CASP Q9.	Do the benefits of the experimental intervention outweigh the harms and costs?	CT	CT
CASP Q10.	Can the results be applied to your local population/in your context?	CT	
CASP Q11.	Would the experimental intervention provide greater value to the people in your care than any of the existing interventions?	CT	CT
CASP Q12.	Appraisal summary	The sample size is too small. It was not clear how participants were allocated to either the face-to-face or telepsychiatry group.	Did not compare the outcomes between telemental health services and in-person services.

Notes: Y = Criteria was met, N = Criteria not met, CT = Cannot tell if criteria was met

In the study by Crowe et al. [25], it was not clear how participants were allocated to the face-to-face group or telepsychiatry group. It appears that the study groups were not similar at the start of the study as at the baseline, only mental health outcomes were compared. The characteristics for each group (e.g. age, sex, etc.) were not reported. The mean score on the coping subscale (as measured by 13-item subscale created from the OMS Instrument) was 26.10 for those who used face-to-face therapy compared to the scores of those who used telepsychiatry (M = 27.56). For psychiatric symptoms (as measured by the 11-item subscale created from the OMS Instrument), M = 18.80 for face-to-face compared to M = 17.50 for the telepsychiatry group. The results of baseline data revealed no significant differences between groups. The effects of intervention were not fully reported because the power calculation was not stated, although the results of the t-test statistical analysis and the p value were. Crowe et al. [25] found no significant difference in coping abilities and psychiatric symptoms between those who used face-to-face psychotherapy and those receiving telepsychiatry, and that both groups reported a high level of satisfaction with regards to service provision. However, the results should be interpreted with caution as the sample size was too small to draw robust conclusions and it was not clear how participants were allocated to the face-to-face or telepsychiatry group. Additionally, the treatment plan within the group and across the groups is not consistent (e.g. variance in session duration).

Pertz et al. [26]’s study included the incorporation of telemental health services tailored to each individual patient in order to assess its feasibility for Deaf patients (n = 50). The use of telemental health versus in-person visits was left to the patient to decide. It is not clear whether there was a comparator. The outcomes measured included the use of PHQ-9 and GAD-7 ASL, Psychosocial Acuity Scale (PAS), and the patient‘s subjective evaluations (which was obtained by outside staff fluent in ASL not involved in the program). Numbers of sessions ranged between 1 and 37 per patient, but it was not clear if that was because of drop out or intensity of treatment or some other unknown reason. Although the PHQ-9 and GAD-7 were used to measure outcomes, results were not compared between those who used in-person vs. telemental health visits. With regards to the feedback on the use of telemental health services, patients praised the option of having an opportunity to receive telemental health care remotely.

## Discussion

The findings from this systematic review have identified that there is a significant evidence gap in the literature concerning videoconferencing telemedicine interventions with Deaf people. The results from this review identified only two studies, and these do not fully answer the review’s research question. Both Crowe et al. [25] and Pertz et al. [26] carried out small scale studies involving the use of telemedicine, but do not report in full the effectiveness of the intervention, nor any comparator results between those who used face-to-face therapy and those who used the telemedicine therapy intervention. Although participant satisfaction is reported as high in both studies, the details about why this is, are limited. Furthermore, the study designs meant that no patients had experience of both conditions of therapy (face-to-face or telemedicine) on which to base comparative views of satisfaction. Details of key potential confounding factors in satisfaction and outcomes were also missing. For example, whether the therapy in ASL was delivered by a Deaf therapist or a hearing signing therapist. This is important, not for reasons of linguistic fluency, but rather to account for effects of cultural matching of patient(s) to therapist(s). In both studies, therapy in both conditions consisted of direct communication in a shared language. Satisfaction of telemedicine therapy delivered through an interpreter for example is excluded but is a potential confounding factor in comparisons of the conditions of therapy and is known to influence therapeutic alliance (regardless of whether face-to-face or remote access is involved). This review did consider studies from health interventions, however, none were identified that met the inclusion criteria for the review.

With Deaf populations being geographically dispersed and the lack of availability of therapists able to meet Deaf people’s cultural and linguistic needs, telemedicine therapies may offer a solution by increasing access and availability of therapy when required. Therapists who are fluent in sign language would be able to offer therapy to Deaf people directly without the need for an interpreter can be considered as cost effective when compared to delivering the service with an interpreter [6]. However, gaps in the research knowledge remain, including the effectiveness of the intervention for Deaf people, such as whether psychological therapy using telemedicine made a difference or not when compared to face-to-face therapy. There is a need for more research in this area, such as qualitative studies which could include interviews with patients on their satisfaction and acceptability of the use of telemedicine. Crowe [27] acknowledges the gaps in guidelines for providing telemedicine psychotherapy for Deaf people. Given the COVID-19 pandemic, more health professionals are using telemedicine to ensure that they are continuing to provide therapies that are accessible for Deaf people, however the knowledge of its effectiveness remains small.

## Conclusion

Given the limited evidence available, it was not possible to reach a conclusion on the efficacy and effectiveness of telemedicine interventions for Deaf people when compared with face-to-face interventions whether in health or mental health. Research into the effectiveness of telemedicine intervention for Deaf people is much needed. The gap in the knowledge is important as it highlights the much-needed research in this area, which would help tackle, both, the inequalities in access to services, and in mental health and health-related outcomes for Deaf people.

## Data Availability

All data generated or analysed during this study are included in this published article.

## References

[1] Kvam M, Loeb M, Tambs K (2016). Mental Health in Deaf adults: symptoms of anxiety and Depression among hearing and Deaf individuals. J Deaf Stud Deaf Educ.

[2] Barnett S, Klein J, Pollard R, Samar V, Schlehofer D, Starr M, Sutter E, Yang H, Pearson T (2011). Community Participatory Research with Deaf sign Language users to identify Health Inequities. Am J Public Health.

[3] Alexander A, Ladd P, Powell S (2012). Deafness might damage your health. The Lancet.

[4] NICE. Common mental health problems: identification and pathways to care. NICE CG123. Leicester, UK: The British Psychological Society and The Royal College of Psychiatrists. 2011. https://www.nice.org.uk/guidance/cg123 [Accessed 09 December 2021].

[5] Joint Commissioning Panel for Mental Health. Guidance for Commissioners of Primary Care Mental Health Services for Deaf People (Psychological Therapies). The Royal College of Psychiatrists. 2017. https://www.jcpmh.info/resource/guidance-commissioners-primary-care-mental-health-services-deaf-people/ [Accessed 16th September 2020].

[6] Young A, Rogers K, Davies L, Pilling M, Lovell K, Pilling S, Belk R, Shields G, Dodds C, Campbell M, Nassimi-Green C, Buck D, Oram R. Evaluating the effectiveness and cost-effectiveness of British Sign Language Improving Access to Psychological Therapies: an exploratory study. Health Serv Delivery Res. 2017;5(24). 28880505

[7] ; SignHealth [Internet]. London: SignHealth, March. 2022. Press release, SignHealth awarded ground-breaking NHS England contract to improve Deaf people’s access to mental health support; 2022 Mar [cited 2022 Jun 15]. Available from: https://signhealth.org.uk/press-release/ground-breaking-nhs-contract-to-improve-deaf-peoples-access-to-mental-health-support/.

[8] Sutton-Spence R, Woll B (1999). The Linguistics of British sign Language: an introduction.

[9] Ladd P (2003). Understanding Deaf Culture: in search of Deafhood.

[10] Atherton M. Choosing to be deaf: leisure and sport in the deaf community of north-west England, 1945–1995. PhD Thesis. Leicester: De Montfort University; 2005. https://dora.dmu.ac.uk/bitstream/handle/2086/4957/thesis%20-%20final%20bound%20version.pdf?sequence=1&isAllowed=y [Accessed 24 May 2022].

[11] BDA. Access to Services for Deaf People. British Deaf Association and Derby Deaf Forum. 2014. https://bda.org.uk/wp-content/uploads/2017/03/BDA_Access_to_Services_for_Deaf_People_Derby_Report_11-2014.pdf [Accessed 24 May 2022].

[12] Walter G. In: Dirmyer R, editor. Number of persons who are Deaf or Hard of hearing: Rochester, NY. National Technical Institute for the Deaf, Rochester Institute of Technology; 2012. https://www.rit.edu/ntid/sites/rit.edu.ntid/files/collaboratory/number_of_persons_who_are_deaf_or_hard_of_hearing_rochester.pdf. [Accessed 24 May 2022].

[13] Rush KL, Howlett L, Munro A, Burton L (2018). Videoconference compared to telephone in healthcare delivery: a systematic review. Int J Med Informatics.

[14] Moore D, Guthmann D, Rogers N, Frake S, Embree J (2009). E-therapy as a Means for addressing barriers to Substance Use Disorder treatment for persons who are Deaf. J Sociol Social Welf.

[15] Belk R, Pilling M, Rogers KD, Lovell K, Young A (2016). The theoretical and practical determination of clinical cut-offs for the british sign Language versions of PHQ-9 and GAD-7. BMC Psychiatry.

[16] James TG, Coady KA, Stacciarini JMR, McKee MM, Phillips DG, Maruca D, Cheong JW (2022). They’re not willing to Accommodate Deaf Patients”: communication experiences of Deaf American sign Language users in the Emergency Department. Qual Health Res.

[17] Yabe M (2020). Healthcare providers’ and deaf patients’ interpreting preferences for critical care and non-critical care: video remote interpreting. Disabil Health J.

[18] Richardson L, Frueh BC, Grubaugh A, Egede L, Elhai J (2009). Current directions in Videoconferencing Tele-Mental Health Research. Clin Psychol Sci Pract.

[19] Flodgren G, Rachas A, Farmer AJ, Inzitari M, Shepperd S. Interactive telemedicine: effects on professional practice and health care outcomes. Cochrane Database of Systematic Reviews. 2015;9. 10.1002/14651858.CD002098.pub2. 10.1002/14651858.CD002098.pub2 PMC6473731 26343551

[20] Gournaris MJ (2019). Preparation for the delivery of Telemental Health Services with individuals who are Deaf: informed consent and Providers Procedure Guidelines. J Am Deafness Rehabilitation Association.

[21] Austen S, McGrath M (2006). Attitudes to the use of videoconferencing in general and specialist psychiatric services. J Telemed Telecare.

[22] Booth A, Fry-Smith A. Developing the research question. In: Topfer L, Auston I, editors. Etext on Health Technology Assessment (HTA) Information Resources. Bethesda, Maryland: National Information Center on Health Services and Health Care Technology; 2003 [cited 2021 Dec 09]. Available from: https://www.nlm.nih.gov/archive/20060905/nichsr/ehta/chapter2.html.

[23] Rogers K, Lovell K, Bower P, Armitage CJ. What are the Deaf sign language users’ experiences as patients in healthcare services? A scoping review protocol. Inplasy Protoc 202210102; 10.37766/inplasy2022.1.0102. 10.1371/journal.pgph.0003535 PMC11864532 40009582

[24] Critical Appraisal Skills Programme [Internet]. CASP Checklist. 2019 [cited 2021 Dec 09]. Available from: https://casp-uk.net/casp-tools-checklists/.

[25] Crowe T, Jani S, Jani S, Jani N, Jani R (2016). A pilot program in rural telepsychiatry for deaf and hard of hearing populations. Heliyon.

[26] Pertz P, Plegue M, Diehl K, Zazove P, McKee M (2018). Addressing Mental Health needs for Deaf Patients through an Integrated Health Care Model. J Deaf Stud Deaf Educ.

[27] Crowe T (2017). Telemental Health Services as a targeted intervention for individuals who are Deaf and Hard of hearing. J Am Deafness Rehabilitation Association.

